# Gβγ subunit signalling underlies neuropeptide Y‐stimulated vasoconstriction in rat mesenteric and coronary arteries

**DOI:** 10.1111/bph.16192

**Published:** 2023-08-08

**Authors:** JinHeng Lin, Lauren Scullion, Christopher J. Garland, Kim Dora

**Affiliations:** ^1^ Department of Pharmacology University of Oxford Oxford UK

**Keywords:** calcium signalling, coronary artery, Gβγ subunit, neuropeptide Y, tension, Y_1_ receptor

## Abstract

**Background and Purpose:**

Raised serum concentrations of the sympathetic co‐transmitter neuropeptide Y (NPY) are linked to cardiovascular diseases. However, the signalling mechanism for vascular smooth muscle (VSM) constriction to NPY is poorly understood. Therefore, the present study investigated the mechanisms of NPY‐induced vasoconstriction in rat small mesenteric (RMA) and coronary (RCA) arteries.

**Experimental Approach:**

Third‐order mesenteric or intra‐septal arteries from male Wistar rats were assessed in wire myographs for isometric tension, VSM membrane potential and VSM intracellular Ca^2+^ events.

**Key Results:**

NPY stimulated concentration‐dependent vasoconstriction in both RMA and RCA, which was augmented by blocking NO synthase or endothelial denudation in RMA. NPY‐mediated vasoconstriction was blocked by the selective Y_1_ receptor antagonist BIBO 3304 and Y_1_ receptor protein expression was detected in both the VSM and endothelial cells in RMA and RCA. The selective Gβγ subunit inhibitor gallein and the PLC inhibitor U‐73122 attenuated NPY‐induced vasoconstriction. Signalling via the Gβγ–PLC pathway stimulated VSM Ca^2+^ waves and whole‐field synchronised Ca^2+^ flashes in RMA and increased the frequency of Ca^2+^ flashes in myogenically active RCA. Furthermore, in RMA, the Gβγ pathway linked NPY to VSM depolarization and generation of action potential‐like spikes associated with intense vasoconstriction. This depolarization activated L‐type voltage‐gated Ca^2+^ channels, as nifedipine abolished NPY‐mediated vasoconstriction.

**Conclusions and Implications:**

These data suggest that the Gβγ subunit, which dissociates upon Y_1_ receptor activation, initiates VSM membrane depolarization and Ca^2+^ mobilisation to cause vasoconstriction. This model may help explain the development of microvascular vasospasm during raised sympathetic nerve activity.

**Abbreviations**
AP: action potential
EC: endothelial cell
EDH: endothelium‐dependent hyperpolarisation
MT: myogenic tone
PE: phenylephrine
PTX: pertussis toxin
RCA: rat coronary artery
RMA: rat mesenteric artery
TTCC: T‐type calcium channels
VGCC: voltage‐gated calcium channels
VSM: vascular smooth muscle

**What is already known**

- NPY is a vasoconstrictor released from sympathetic nerves; elevated serum NPY links to cardiovascular disease.

**What does this study add**

- This study identifies a link between the Gβγ subunit and small artery vasoconstriction to NPY.

**What is the clinical significance**

- NPY activity may contribute to microvascular spasm.
- Targeting vascular Y_1_ receptors offers a potential therapeutic option for treating coronary microvascular dysfunction.

## 1 INTRODUCTION

The sympathetic co‐transmitter neuropeptide Y (NPY) is released following periods of high sympathetic drive and plays an important role in regulating cardiovascular function (Tan et al., 2018). Elevated levels of NPY are implicated in a range of cardiovascular diseases including stress‐induced cardiomyopathy (Dvorakova et al., 2014), heart failure (Ajijola et al., 2020) and myocardial infarction (Herring et al., 2019). Notably, in patients with heart failure and with ST‐elevated myocardial infarction (STEMI), high serum NPY levels are linked to poor coronary microvascular function, arrhythmia, hypertension, impaired recovery of cardiac function and death (Ajijola et al., 2020; Herring et al., 2019). Although NPY can exert a direct effect on cardiomyocytes (Heredia et al., 2005), poor prognosis in patients with high NPY levels is likely, at least in part, to be the result of coronary microvascular dysfunction, as high microcirculatory resistance in STEMI patients following intervention predicts poor long‐term cardiac outcomes (Fearon et al., 2013).

Since its discovery in the late 1980s, the vasoactive properties of NPY have been widely studied. When infused in vivo, NPY increases blood pressure and decreases regional blood flow (Bischoff et al., 2021; Malmstrom et al., 1997). In ex vivo studies with isolated arteries, NPY is a vasoconstrictor in a variety of species across different vascular beds (Nilsson et al., 1996; Prieto et al., 2000; Xia et al., 1992), including coronary arteries of rat and human (Herring et al., 2019; Prieto et al., 1998; Tseng et al., 1988). Interestingly, however, there is conflicting evidence as to whether NPY increases intracellular Ca^2+^ levels [Ca^2+^]_*i*_ in vascular smooth muscle (VSM) cells in these arteries during constriction (Herring et al., 2019; Jacques et al., 2000; Mihara et al., 1989; Prieto et al., 2000; Wier et al., 2009). Moreover, while it is recognised that all NPY receptors couple to G_i/o_ (Alexander, Christopoulos et al., 2021), the possible downstream pathways leading to Ca^2+^ mobilisation and VSM constriction remain poorly characterised.

The present study investigated the signalling mechanisms underlying NPY‐induced vasoconstriction in two resistance arteries, rat mesenteric and coronary arteries. We hypothesised that VSM NPY Y_1_ receptor activation may lead to vasoconstriction through a Gα_i/o_‐independent, Ca^2+^‐dependent pathway.

## 2 METHODS

### 2.1 Animals

The use of male Wistar rats (210–320 g, Charles River Laboratories) was approved by the University of Oxford ethical committee. These studies comply with the latest ARRIVE guidelines (Percie du Sert et al., 2020) and updated recommendations from the BJP (Lilley et al., 2020). Animals were housed in individually ventilated cages, in a temperature‐controlled environment with a 24‐h light–dark cycle, with food pellets and water ad libitum. Following delivery, animals were allowed to acclimatise for at least 10 days before use. Rats were killed by exposure to rising concentrations of CO_2_ for 3 min, and, following the loss of the righting reflex, death was confirmed by cervical dislocation, in compliance with the Animals (Scientific Procedures) Act 1986.

### 2.2 Experimental design and group sizes

Minimum group sizes were determined through a priori power calculations, performed with G*Power software (v3.1.9.6, open source; Faul et al., 2007), and were increased to at least *n* = 5 if calculated group sizes were smaller than five. Sample size was calculated at *α* = 0.05, power = 0.8, 15% SD and effect size estimated as ability to detect 50% difference from control. Group sizes were designed to be equal, with any extra biological replicates being added if excess arteries were available on the day, prioritising same‐day control experiments.

Randomisation and blinding were not performed as they were impractical and would require additional personnel. Different pharmacological agents had to be clearly labelled and had different dilution factors for final concentrations. Furthermore, lack of randomisation and blinding was unlikely to skew the results as multiple arteries from one animal were used for different treatment protocols.

### 2.3 Reagents and antibodies

Phenylephrine (PE; P6126), acetylcholine (ACh; A6625), *N* ^ω^‐nitro‐l‐arginine methyl ester (l‐NAME; N5751), pertussis toxin (PTX; P7208) and nifedipine (N7634) were purchased from Merck (Gillingham, UK). NS 6180 (4864), apamin (1652), BIBO 3304 (2412), gallein (3090), [Leu^31^,Pro^34^]‐NPY (1176), NNC 55‐0396 (2268) and U‐73122 (1268) were purchased from Tocris (Abingdon, UK). NPY‐(3‐36) (049‐27) and [D‐Trp34]‐NPY (049‐24) were from Phoenix Pharmaceuticals (Karlsruhe, Germany), and NPY (AS‐22465) was from Eurogentec (Seraing, Belgium). BIBO 3304, gallein and NNC 55‐0396 were dissolved in DMSO, and nifedipine was dissolved in 100% ethanol. All other drugs and peptides were dissolved in water. Final solvent concentrations were below 0.2% for all experiments.

Primary antibodies were as follows: 1:200 rabbit polyclonal anti‐human NPY_1_ receptor IgG (aa 350 to C‐terminus; ab183108, Abcam, Cambridge, UK; RRID:AB_2921334), 1:200 sheep polyclonal anti‐rat tyrosine hydroxylase IgG (full length protein; ab113, Abcam; RRID:AB_297905), 1:1000 mouse monoclonal anti‐human NPY IgG (aa 29‐64; ab112473, Abcam; RRID:AB_10861167) and 1:200 mouse monoclonal anti‐*Bordetella pertussis* toxin IgG (S1 subunit; sc‐57639, Santa Cruz, Dallas, USA; RRID:AB_781659). Secondary antibodies were as follows: 1:1000 Alexa Fluor 488 goat anti‐rabbit IgG (A11034, Invitrogen, Paisley, UK), 1:1000 Alexa Flour 546 donkey anti‐sheep IgG (A21098, Invitrogen) and 1:1000 Alexa Fluor 647 goat anti‐mouse IgG (A21235, Invitrogen). Nuclei were stained with 1:10,000 Hoechst 33342 (H3570, Invitrogen).

### 2.4 Wire myography

The heart and the small intestines with intact mesentery were excised from rats and placed in cold Krebs solution containing (in mM): 121.3 NaCl, 25 NaHCO_3_, 4.7 KCl, 1.2 MgSO_4_·7H_2_O, 1.2 KH_2_PO_4_, 1.25 CaCl_2_ and 11.6 glucose. Rat mesenteric arteries (RMAs; third‐order mesenteric artery, 100–330 μm) and rat coronary arteries (RCAs; septal artery, 180–360 μm) were dissected free of connective tissue, fat and cardiomyocytes, and 1–2 mm segments were mounted in a wire myograph chamber (model 610M, Danish Myo Technology A/S, Hinnerup, Denmark), linked to a PowerLab data acquisition system (model 4/20, AD Instruments, Oxford, UK) and LabChart software (v8.1.17, AD Instruments).

Arteries were incubated in Krebs solution gassed with 21% O_2_, 5% CO_2_, in N_2_. The solution temperature was raised to 37°C, and the artery was stretched and normalised to a resting tension equivalent to that generated at 90% of the diameter of the vessel at 70 (RMA) or 80 (RCA) mmHg (Mulvany & Halpern, 1977). Following an equilibration period of 30 min, endothelial function was assessed by >90% relaxation to 300 nM ACh from pre‐constriction with phenylephrine (PE), and only arteries with viable endothelium were used for further study. Concentration–response curves (CRCs) for vasoconstriction induced by NPY, or its analogues, were performed in half‐log increments (0.1–300 nM; 100 s intervals), following incubation with various combinations of pharmacological inhibitors (5 min for nifedipine; 30 min for l‐NAME, NS 6180, BIBO 3304, gallein, NNC 55‐0396; 1 h for apamin, U‐73122; 1 or 3 h for PTX). Only one NPY CRC was performed per artery due to receptor desensitisation. NPY‐induced constriction was normalised as a percentage of the constriction stimulated by isotonic 45 mM K^+^ solution, to control for the GPCR‐independent contractile capacity of each individual artery.

In a subset of experiments using endothelium‐denuded (without endothelial cells: −EC) arteries, denudation was performed by passing a human hair through the lumen of the artery and gently rubbing the lumen. Arteries with <10% relaxation to 1 μM ACh from pre‐constriction with PE were considered denuded. NPY‐induced constriction in −EC arteries were normalised to 45 mM K^+^ constriction after denudation.

### 2.5 Immunohistochemistry

Upon completion of tension measurements, RMA and RCA were fixed in situ in wire myography chambers with 4% paraformaldehyde for 1 h at room temperature and washed with PBS. Fixed arteries were sliced open laterally and removed from the wire myograph and blocked for 1 h at room temperature with blocking buffer (1% BSA, 0.5% Triton X‐100, 0.05% Tween 20 in PBS). Arteries were then incubated with the primary antibodies overnight at 4°C, followed by secondary antibodies and Hoechst for 2 h at room temperature. Antibodies and Hoechst were diluted in blocking buffer. After labelling was complete, the opened arteries were carefully placed in mounting medium on glass coverslips such that the flat endothelial cell (EC) layer faced the coverslip. The Immuno‐related procedures used comply with the recommendations made by the *British Journal of Pharmacology* (Alexander et al., 2018).

Arteries were excited at 405, 488, 543 and 635 nm; the fluorescence emitted at 430–470, 505–540, 560–620 and 655–755 nm was acquired through a water immersion objective (1.15 NA, 1024 × 1024 pixels; Olympus) using a laser scanning confocal microscope (FV1200; Olympus, Southend‐on‐Sea, UK). Sequential z‐stacks through the artery wall were obtained at 1 μm increments using Fluoview Software (FV10‐ASW 3.0; Olympus) and reconstructed in Imaris Software (version 8.0.2; Bitplane).

### 2.6 VSM Ca^2+^ imaging

RMA and RCA were mounted and normalised in a confocal wire myograph (model 120CW, Danish Myo Technology A/S), and an endothelial function test was performed, as described above in the *wire myography* section. Viable arteries were loaded with the calcium‐sensitive fluorescent dye Calbryte 520 AM (20650, AAT Bioquest, Pleasanton, USA) (2.5 μM; dissolved in DMSO and 0.03% [w/v] Pluronic F‐127) for 30 min at 30°C, then incubated in Krebs buffer for 30 min at 37°C to allow de‐esterification. After excitation at 488 nm, the fluorescence emission intensity at 513–563 nm was recorded from the bottom surface of arteries using a spinning disc confocal microscope (Yokogawa CSU22) fitted with an Andor (Abingdon, UK) iXON DV887ECS‐BV camera mounted on an Olympus IX70 inverted microscope using a water immersion objective (×40, aperture 0.8, working distance 3.3 mm; Olympus), as performed previously (Smith et al., 2020). Images (430 × 420 pixels, 35 Hz) were stored for offline analysis (iQ version 3.5, Andor Bioimaging Division; MetaMorph version 7.7.4.0, Molecular Devices, San Jose, USA).

Following background subtraction, average relative changes in [Ca^2+^] were calculated as changes in intensity of fluorescence divided by fluorescence at time 0 s (F/F_0_), within selected cell regions (for Ca^2+^ waves; five individual cells selected per experiment) or the whole field (Ca^2+^ flashes).

### 2.7 Electrophysiology in isometrically tensioned arteries

Isolated RMA were mounted in a wire myograph for simultaneous measurement of VSM membrane potential (*V*_m_) and isometric tension, as previously described (Smith et al., 2020). After normalisation and endothelial function test, VSM membrane potential and tension were recorded through a pre‐amplifier (Neurolog system, Digitimer Ltd., Welwyn Garden City, UK) linked to a MacLab data acquisition system (model 4e, AD Instruments) and LabChart software (v8.1.17 AD Instruments). Individual VSM cells were impaled with sharp glass microelectrodes (backfilled with 2 M KCl; tip resistances approximately 60 MΩ), observed as a rapid deflection towards the resting membrane potential, near −50 mV.

### 2.8 Data and statistical analysis

NPY CRCs were fitted using nonlinear regression in GraphPad Prism (v9.4.0, GraphPad Software, Boston, USA). *E*_max_ was determined as the highest point on the CRC, usually at 100 or 300 nM NPY. Ca^2+^ imaging data are expressed as the frequency of Ca^2+^ events observed per second (Hz), quantified within a 20 s duration in the section immediately after initiation of waves (for Ca^2+^ waves), or at the peak of NPY constriction (for Ca^2+^ flashes). Electrophysiology data are presented as change in *V*_m_ (Δ*V*_m_) from baseline following stimulation with 100 nM NPY, and the frequency and amplitude of action potential (AP) spikes were quantified at the peak (20 s duration) of NPY constriction.

The data and statistical analysis comply with the BJP recommendations on experimental design and analysis (Curtis et al., 2022). Averaged data in all graphs are expressed as the mean ± SEM. *n* denotes biological replicates; each *n* within the same group represents an artery derived from a different animal. In experiments where technical replicates were performed, these were averaged and reported as *n* = 1 in statistical analysis. The same sets of control wire myography experiments (blank squares, without l‐NAME; filled black circles, with l‐NAME) were used in multiple figures, as same‐day control was not feasible. Statistical analysis was performed using GraphPad Prism, with statistical significance (*) indicated by an alpha value of *P* < 0.05; the type of analysis for each dataset is indicated within the respective figure legend. Parametric analysis was performed only if all datasets passed the Shapiro–Wilk normality test. Post‐hoc tests were run only if F achieved *P* < 0.05 and there was no significant variance inhomogeneity. Brown–Forsythe and Welch ANOVA, with Dunnett's T3 multiple comparisons test, was performed to adjust for unequal SDs in datasets that tested significant for the Brown–Forsythe test.

### 2.9 Nomenclature of targets and ligands

Key protein targets and ligands in this article are hyperlinked to corresponding entries in http://www.guidetopharmacology.org and are permanently archived in the Concise Guide to PHARMACOLOGY 2021/22 (Alexander, Christopoulos et al., 2021; Alexander, Fabbro et al., 2021; Alexander, Kelly et al., 2021; Alexander, Mathie et al., 2021).

## 3 RESULTS

### 3.1 Endothelial regulation of NPY‐induced vasoconstriction

**FIGURE 1:**
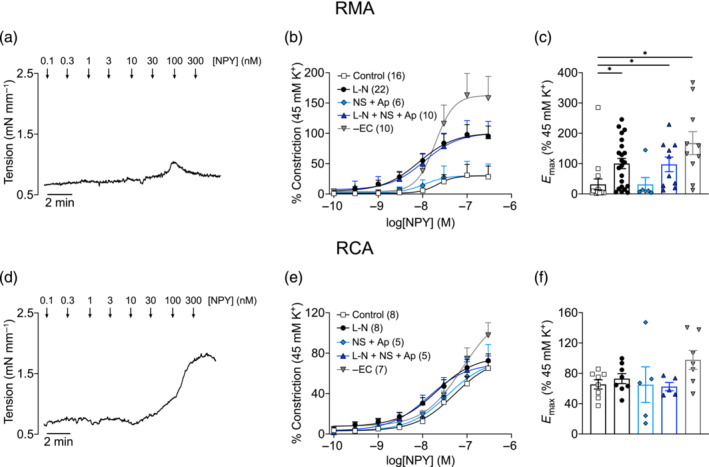
Endothelial regulation of NPY‐induced vasoconstriction in rat mesenteric artery (RMA) (top row) and rat coronary artery (RCA) (bottom row). (a,d) Representative wire myograph traces of vasoconstriction stimulated by NPY (0.1–300 nM) in (a) RMA and (d) RCA under control conditions. (b,e) Concentration–response curves of NPY‐induced constriction in arteries without pharmacological intervention (control) or in the presence of l‐NAME (100 μM, 30 min), NS 6180 (1 μM, 30 min) + apamin (100 nM, 1 h) or the combination of all three drugs. A subset of experiments was performed with endothelium‐denuded (−EC) arteries without additional pharmacological intervention. Vasoconstriction was normalised to the response elicited by 45 mM K^+^ Krebs solution. *n* is indicated in parentheses. (c,f) Summary of the *E*_max_ elicited by NPY from experiments in (b) and (e). Kruskal–Wallis test, with Dunn's multiple comparisons test against control, was performed for (c). Ordinary one‐way ANOVA was performed for (f), without post hoc analysis as ANOVA was not significant. * *P* < 0.05.

NPY has been shown to be a weak vasoconstrictor on its own in RMA (Cortes et al., 1999). In wire myography experiments with isolated RMA, 75% (of *n* = 16) of vessels studied under control conditions constricted minimally (*E*_max_ < 10%; Figure 1a) in response to increasing concentrations of NPY, up to 300 nM. On average, the *E*_max_ of control NPY response in RMA was 31.4 ± 17.9% (Figure 1b,c). On the other hand, RCA constricted more consistently to NPY challenge, as all vessels constricted >35% to NPY (Figure 1d–f; *E*_max_ 65.3 ± 6.1%). Additionally, significantly larger constriction to NPY was observed in endothelium‐denuded RMA (Figure 1b,c; *E*_max_ 167.5 ± 37.9%), but not RCA (Figure 1e,f; *E*_max_ 97.7 ± 12.4%; post hoc analysis not performed).

To investigate the role of the endothelium in modulating NPY‐mediated vasoconstriction, arteries were pre‐incubated with l‐NAME, NS 6180 + apamin (inhibitors of K_Ca_ channels facilitating endothelium‐dependent hyperpolarisation [EDH]) or the combination of all three, prior to stimulation with NPY. Blocking eNOS and EDH with these agents did not stimulate measurable myogenic tone (MT) in RMA, whereas there was a trend that MT was higher in RCA treated with l‐NAME and with all three blockers (Figure S1). Following l‐NAME treatment, the constriction to NPY was significantly larger in RMA (*E*_max_ 100.3 ± 16.7%), but not RCA (*E*_max_ 73.1 ± 6.6%), compared with untreated arteries. In contrast, blocking EDH with NS 6180 and apamin did not augment RMA (*E*_max_ 31.3 ± 22.7%) or RCA (*E*_max_ 65.0 ± 23.6%) constriction to NPY. Similar to pre‐treatment with l‐NAME only, incubation with all three blockers significantly enhanced the vasoconstriction in RMA (*E*_max_ 97.4 ± 24.0%), but not in RCA (*E*_max_ 62.5 ± 5.5%). To facilitate consistency of NPY responses, especially for RMA, vessels were pre‐treated with l‐NAME for all subsequent wire myography experiments.

### 3.2 Expression and functional coupling of the NPY Y_1_ receptor

**FIGURE 2:**
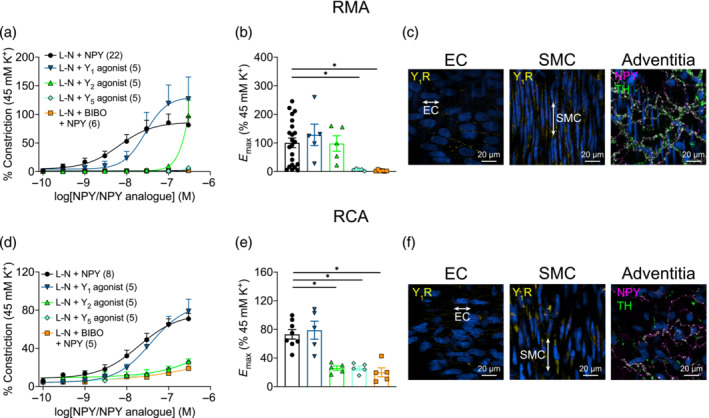
The Y_1_ receptor mediates NPY‐activated vasoconstriction in rat mesenteric artery (RMA) (top row) and rat coronary artery (RCA) (bottom row). (a,d) Concentration–response curves of NPY‐induced constriction in arteries pre‐treated with l‐NAME (L‐N), with or without BIBO 3304 (1 μM, 30 min). Additionally, in place of NPY, specific agonists for Y_1_ ([Leu^31^,Pro^34^]‐NPY), Y_2_ (NPY‐(3‐36)) or Y_5_ ([D‐Trp34]‐NPY) receptors were used to construct CRCs. Vasoconstriction was normalised to the response elicited by 45 mM K^+^ Krebs solution. *n* is indicated in parentheses. (b,e) Summary of the *E*_max_ elicited by NPY from experiments in (a) and (d). Brown–Forsythe and Welch ANOVA test with Dunnett's T3 multiple comparisons test against L‐N + NPY was performed for both (b) and (e). (c,f) Representative ([c] *n* = 6 for RMA and [f] *n* = 7 for RCA) immunohistochemistry labelling for the Y_1_ receptor (yellow) in the smooth muscle cells and endothelial cells and NPY (magenta) and tyrosine hydroxylase (green) labelling sympathetic nerves in the adventitia; white signal indicates co‐localisation of NPY and tyrosine hydroxylase. Nuclei were labelled in blue. * *P* < 0.05.

We have previously reported that NPY‐induced vasoconstriction was inhibited by BIBO 3304, an NPY Y_1_ receptor selective antagonist (Wieland et al., 1998), in pressurised RCA (Herring et al., 2019). This was replicated here in the wire myography set‐up, whereby BIBO 3304 abolished the vasoconstriction in both RMA (Figure 2a,b; *E*_max_ 2.8 ± 1.0%) and RCA (Figure 2d,e; *E*_max_ 20.0 ± 6.2%). The major involvement of Y_1_ receptor activation in vasoconstriction is also supported by experiments using NPY receptor subtype‐specific agonists. The Y_1_ receptor‐selective agonist [Leu^31^,Pro^34^]‐NPY (Fuhlendorff et al., 1990) induced a similar vasoconstrictive effect as NPY in both RMA (*E*_max_ 128.3 ± 37.7%; n.s. vs. L‐N + NPY) and RCA (*E*_max_ 78.8 ± 12.6%; n.s. vs. L‐N + NPY). On the other hand, the maximal vasoconstriction produced by the Y_2_ receptor‐selective agonist NPY‐(3‐36) (Gehlert et al., 1996; Grandt et al., 1996) was significantly smaller than that by NPY in RCA (*E*_max_ 25.9 ± 3.0%), but not RMA (*E*_max_ 97.9 ± 27.2%). Notably, however, the CRC for NPY‐(3‐36) in RMA was markedly right‐shifted (EC_50_ = 441.3 nM for NPY‐(3‐36) vs. 10.7 nM for NPY), suggesting that the mesenteric vascular Y_2_ receptor may be activated only at high NPY concentrations. Lastly, the Y_5_ receptor‐selective [D‐Trp^34^]‐NPY (E. M. Parker et al., 2000) also induced significantly less constriction in both RMA (*E*_max_ 6.4 ± 1.6%) and RCA (*E*_max_ 25.7 ± 3.0%).

Immunohistochemistry was performed to establish the expression of the functionally important NPY Y_1_ receptor in RMA and RCA. Discrete expression of the Y_1_ receptor was detected in the VSM of both RMA and RCA (Figure 2c,f; negative control presented in Figure S2). Interestingly, punctate expression of the Y_1_ receptor was also detected in a portion of endothelial cells in both vessel types. The expression and distribution of endogenous NPY was also confirmed, alongside the sympathetic nerve marker tyrosine hydroxylase, in the adventitia, but not the EC and SMC layers, of the arteries.

### 3.3 Role of Gβγ and PLC in NPY‐induced vasoconstriction

To investigate signalling immediately downstream of Y_1_ receptor activation, the selective inhibitor of the G_i/o_ protein family PTX, the selective inhibitor of the Gβγ subunit gallein (Lehmann et al., 2008) and the PLC inhibitor U‐73122 were utilised. Pre‐treatment with PTX did not block NPY‐induced vasoconstriction in RMA (Figure S3A,B), most likely due to inadequate permeation of the toxin into the smooth muscle layer (Figure S3C,D).

**FIGURE 3:**
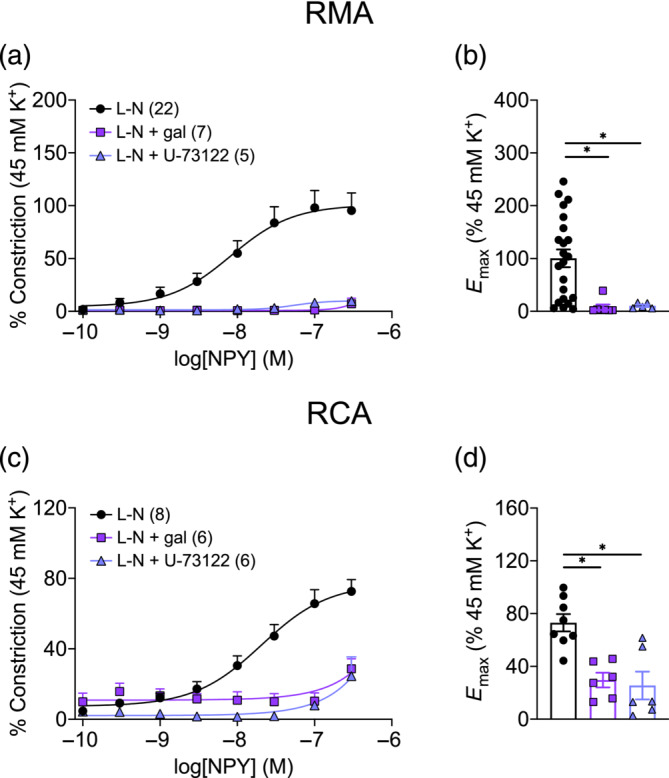
NPY‐induced vasoconstriction is dependent on the Gβγ subunit in (a,b) rat mesenteric artery (RMA) and (c,d) rat coronary artery (RCA). (a,c) Concentration–response curves of NPY‐induced constriction in arteries pre‐treated with l‐NAME (L‐N), with or without gallein (100 μM, 30 min) or U‐73122 (3 μM, 60 min). Vasoconstriction was normalised to the response elicited by 45 mM K^+^ Krebs solution. *n* is indicated in parentheses. (b,d) Summary of the *E*_max_ elicited by NPY from experiments in (a) and (c). Brown–Forsythe and Welch ANOVA test with Dunnett's T3 multiple comparisons test against the L‐N group was performed for (b), while Kruskal–Wallis test with Dunn's multiple comparisons test against the L‐N group was performed for (d). * *P* < 0.05.

Pre‐incubation with gallein significantly attenuated NPY‐induced vasoconstriction in both RMA (Figure 3a,b; *E*_max_ 7.7 ± 5.2%) and RCA (Figure 3c,d; *E*_max_ 29.6 ± 5.6%), each in the presence of l‐NAME, and also in arteries not exposed l‐NAME (Figure S4). Preliminary experiments showed that inhibition of NPY constriction by gallein was concentration dependent, with 100 μM gallein providing consistent and reliable block (Figure S5A). This effect was specific against NPY, as gallein did not inhibit contraction to isotonic 45 mM K^+^ Krebs solution (Figure S5B) or the G_q_‐coupled agonist PE (Figure S5C). Similarly, 1 h incubation with U‐73122, which reduced PE‐induced vasoconstriction by 68.9% but did not inhibit contraction to 45 mM K^+^ (Figure S6), significantly reduced NPY vasoconstriction in both RMA (*E*_max_ 10.5 ± 2.3%) and RCA (*E*_max_ 25.5 ± 10.5%).

### 3.4 NPY activates VSM Ca^2+^ flashes

**FIGURE 4:**
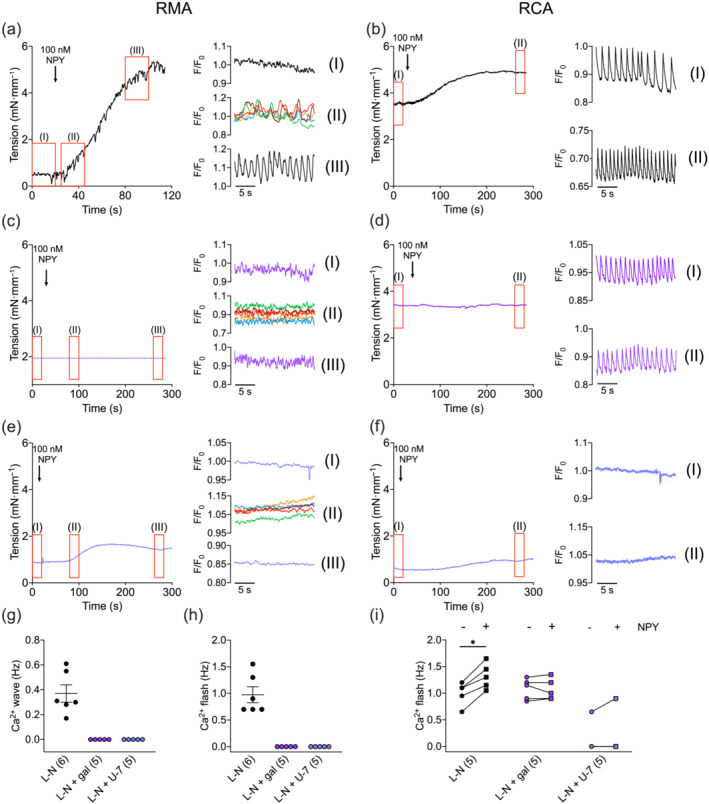
NPY stimulates Ca^2+^ events in rat mesenteric artery (RMA) (left panels) and rat coronary artery (RCA) (right panels). (a–f) Simultaneous measurements of isometric tension and Ca^2+^ events in response to 100 nM NPY in RMA and RCA pre‐treated with l‐NAME (L‐N). Experiments in (c) and (d) were in the presence of gallein (100 μM, 30 min). Experiments in (e) and (f) were in the presence of U‐73122 (3 μM, 60 min). The traces for Ca^2+^ events are magnified, corresponding to sections indicated by red boxes on the tension traces. (aII), (cII) and (eII) follow fluorescence changes in five individual cells (Ca^2+^ waves), whereas other traces were Ca^2+^ changes in the whole field of imaging (Ca^2+^ flashes). (g,h) Frequency of Ca^2+^ waves and flashes activated by NPY in RMA. (i) Changes in the frequency of Ca^2+^ flashes induced by NPY in RCA. *n* is indicated in parentheses. Paired *t* tests were performed on frequency before and after NPY treatment for the L‐N and the L‐N + gal groups, whereas Wilcoxon matched‐pairs signed rank test was performed for the L‐N + U‐7 group. * *P* < 0.05.

Mobilisation of intracellular Ca^2+^ is an essential process in initiating VSM constriction. To study the effects of NPY on VSM Ca^2+^ signalling, arteries were loaded with the Ca^2+^ indicator Calbryte 520 AM, enabling rapid changes in VSM [Ca^2+^]_*i*_ to be imaged using a spinning disc confocal microscope. Representative images of the indicator‐loaded SMCs are shown in Figure S7. In RMA, 100 nM NPY‐activated asynchronous Ca^2+^ waves in individual VSM cells (Figure 4aII,g; 0.37 ± 0.07 Hz). After a delay of 87 ± 14 s, the whole imaging field of RMA VSM began to flash in synchronicity (Figure 4aIII,h; 0.98 ± 0.15 Hz). These Ca^2+^ waves and flashes were completely abolished after pre‐treatment with gallein and U‐73122 (Figure 4c,e,g,h; 0 Hz for both waves and flashes, for both treatment groups). This effect was specific for NPY, as gallein did not block Ca^2+^ flashes to PE (Figure S5D).

RCAs were myogenic and Ca^2+^ flashes occurred before stimulation with NPY (Figure 4bI; 1.00 ± 0.10 Hz). Gallein did not reduce MT (Figure S5E) or lower the frequency of Ca^2+^ flashes (Figure 4dI; 1.08 ± 0.09 Hz), but U‐73122 abolished the flashes in 4/5 of the experiments (Figure 4fI; 0.13 ± 0.13 Hz). Ca^2+^ waves in individual VSM cells were not detected as they were most likely masked by the synchronised Ca^2+^ flashes. NPY (100 nM) significantly increased the frequency of Ca^2+^ flashes (Figure 4bII,i; 1.32 ± 0.11 Hz) in control arteries, but not in the groups pre‐treated with gallein (Figure 4dII,i; 1.07 ± 0.09 Hz) or U‐73122 (Figure 4fII,i; 0.18 ± 0.18 Hz).

### 3.5 NPY depolarizes VSM and activates AP‐like spikes

**FIGURE 5:**
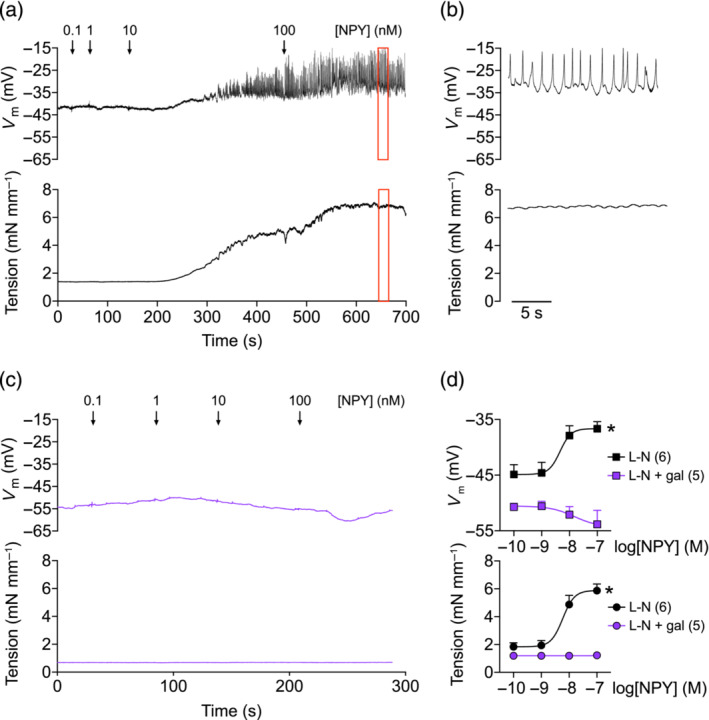
NPY depolarizes rat mesenteric artery (RMA) vascular smooth muscle and activates action potential spikes. (a,c) Simultaneous measurements of isometric tension and *V*_m_ in response to NPY (0.1–100 nM) in RMA pre‐treated with l‐NAME (L‐N), in the (a) absence or (c) presence of 100 μM gallein. (b) Magnified traces of sections indicated by red boxes from (a). (d) Concentration–response curves of NPY‐induced constriction and changes in *V*_m_. *n* is indicated in parentheses. Unpaired *t* tests were performed on the *E*_max_ of both *V*_m_ and tension. * *P* < 0.05.

To further dissect the downstream consequences of VSM Ca^2+^ release events, we measured changes in membrane potential (*V*_m_) of RMA VSM through microelectrode impalement. NPY treatment led to concentration‐dependent depolarization of VSM (Figure 5a,d; Δ*V*_m_ 8.9 ± 1.6 mV), alongside simultaneous vasoconstriction (*E*_max_ 7.0 ± 0.8 mN·mm^−1^). Interestingly, at high NPY concentrations (10–100 nM), the arteries developed transient, AP‐like spikes (Figure 5a,b). At the peak of vasoconstriction, the mean amplitude of these spikes was 18.0 ± 4.3 mV, at a frequency of 1.1 ± 0.2 Hz. The constriction and depolarization to NPY was abolished in arteries pre‐incubated with gallein; instead, NPY caused a slight hyperpolarisation at higher concentrations (Figure 5c,d; Δ*V*_m_ −3.5 ± 2.1 mV).

### 3.6 Involvement of voltage‐gated calcium channels (VGCC)

**FIGURE 6:**
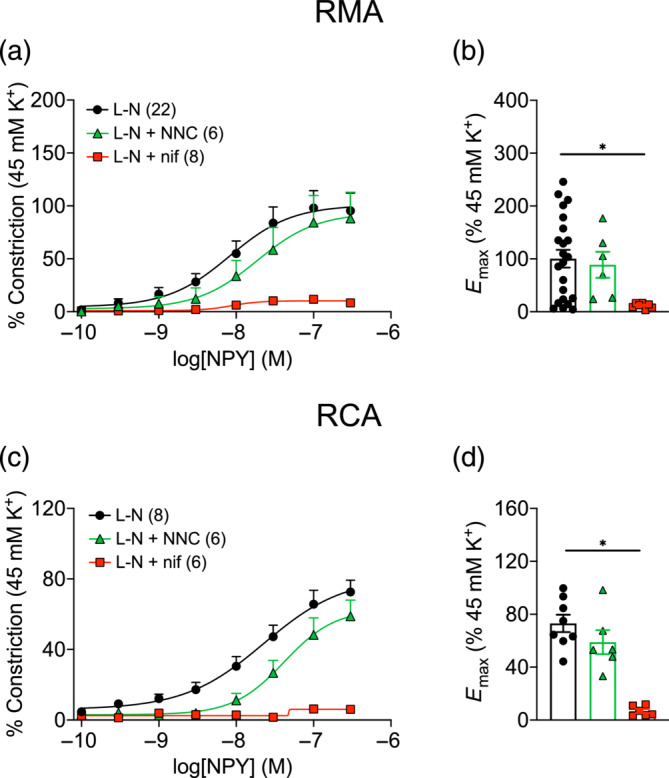
NPY‐induced vasoconstriction is dependent on voltage‐gated calcium channels (VGCC) in (a,b) rat mesenteric artery (RMA) and (c,d) rat coronary artery (RCA). (a,c) Concentration–response curves of NPY‐induced constriction in arteries pre‐treated with l‐NAME (L‐N), with or without NNC 55‐0396 (300 nM, 30 min) or nifedipine (1 μM, 5 min). Vasoconstriction was normalised to the response elicited by 45 mM K^+^ Krebs solution. *n* is indicated in parentheses. (b,d) Summary of the *E*_max_ elicited by NPY from experiments in (a) and (c). Brown–Forsythe and Welch ANOVA test with Dunnett's T3 multiple comparisons test against the L‐N group was performed for (b). Ordinary one‐way ANOVA, with Dunnett's multiple comparisons test against the L‐N group, was performed for (d). * *P* < 0.05.

To examine VGCC involvement, nifedipine and NNC 55‐0396, which selectively block L‐type (LTCC) and T‐type Ca^2+^ channels (TTCC), respectively, were applied. Pre‐treatment with nifedipine abolished NPY vasoconstriction in RMA (Figure 6a,b; *E*_max_ 11.8 ± 1.7%) and in RCA (Figure 6c,d; *E*_max_ 6.8 ± 1.5%). On the other hand, NNC 55‐0396 did not alter responses to NPY in RMA (*E*_max_ 88.8 ± 24.6%; n.s. vs. L‐N) or RCA (*E*_max_ 58.9 ± 9.1%; n.s. vs. L‐N).

## 4 DISCUSSION

The present study provides novel insights into the mechanisms activated by NPY that lead to vasoconstriction in small mesenteric and coronary arteries. Acting on Y_1_ receptors, NPY initiates arterial vasoconstriction through the critical involvement of the Gβγ subunit to activate PLC, mobilise intracellular Ca^2+^ and depolarize the VSM. Depolarization opens VGCCs to facilitate Ca^2+^ influx, further leading to synchronised Ca^2+^ flashes and AP spikes to sustain vasoconstriction. To our knowledge, this is the first report linking Gβγ downstream of G_i/o_‐coupled NPY receptor activation to vasoconstriction, and the first to characterise the transient AP spikes and synchronised Ca^2+^ flashes in response to NPY, in intact ex vivo small arteries. Additionally, there were marked differences in the profile of NPY vasoconstriction in the two vascular beds, summarised in Table 1.

**TABLE 1:** Differences in NPY‐induced vasoconstriction and Ca^2+^ events between RMA and RCA.

RMA	RCA
NPY vasoconstriction significantly augmented by l‐NAME and denuding the endothelium.	NPY vasoconstriction not significantly affected by l‐NAME and denuding the endothelium.
Y_2_‐selective agonist induced significant vasoconstriction at 300 nM.	Y_2_‐selective agonist did not induce significant vasoconstriction.
NPY vasoconstriction completely abolished by BIBO 3304, gallein and U‐73122.	NPY vasoconstriction significantly inhibited, but not abolished, by BIBO 3304, gallein and U‐73122.
More densely innervated by NPY^+^ nerves.	Less densely innervated by NPY^+^ nerves.
Asynchronous Ca^2+^ waves initiated by NPY.	Ca^2+^ waves not measurable, likely masked by Ca^2+^ flashes.
Ca^2+^ flashes activated by NPY.	Spontaneous Ca^2+^ flashes present before stimulation with NPY and frequency increased by NPY.

In the presence of an intact endothelium, a few groups have reported NPY as a weak vasoconstrictor of rat and mouse mesenteric arteries (Cortes et al., 1999; Gonzalez‐Montelongo & Fountain, 2021). Corroborating findings by Cortes et al. (1999), we demonstrate that denuding the endothelium significantly enhanced NPY‐induced vasoconstriction in RMA. Furthermore, our results indicate that NO, but not EDH, plays a major role in modulating NPY‐induced vasoconstriction in RMA. Notably, however, the potentiation of NPY responses by denuding the endothelium and by l‐NAME in RCA was not statistically significant, consistent with our observation that Y_1_ receptor expression was less prevalent in the ECs of RCAs. Evidence supports the expression of NPY receptors in ECs (Sanabria & Silva, 1994; Zukowska‐Grojec et al., 1998), including Y_1_ receptor that we show here in both RMA and RCA, and there is functional evidence of NO‐dependent and ‐independent relaxation by NPY in a number of vascular beds (Nilsson et al., 2000; You et al., 2001). NPY signalling on the ECs thus appears to be vasodilatory, suggesting that in patients with low NO bioavailability and damaged endothelium, common hallmarks of cardiovascular diseases, circulating NPY could be more vasoactive, contributing to hypertension and reduction in coronary blood flow. The role of endothelial NPY receptors on modulating MT warrants further investigation.

Our experiments utilising agonists that preferentially activate different NPY receptor subtypes, and the selective blocker BIBO 3304 (Wieland et al., 1998), demonstrate that NPY‐induced vasoconstriction in RMA and RCA is mediated primarily through Y_1_ receptors, consistent with a number of previous studies pointing to the Y_1_ receptor as the predominant vasoactive receptor subtype (Herring et al., 2019; Nilsson et al., 1996; Prieto et al., 1998). The expression of the Y_1_ receptor mRNA (Nilsson et al., 1996) in intact arteries, and protein (Gonzalez‐Montelongo & Fountain, 2021; Herring et al., 2019) in the VSM of mouse mesenteric and human coronary arteries, have been previously reported; here, we also show that the Y_1_ receptor protein is specifically expressed in both the VSM and the endothelial cells of RMA and RCA. However, a marked vasoconstriction was activated by 300 nM NPY‐(3‐36) in RMA, suggesting a potential vasoactive role for the Y_2_ receptor in these vessels. Indeed, You et al. (2001) have shown that NPY‐(3‐36) significantly constricted rat cerebral arteries, and Tessel et al. (1993) reported that NPY‐(3‐36) was equipotent with [Leu^31^,Pro^34^]‐NPY in constricting rat femoral arteries. In the initial cloning study, the binding affinity of NPY‐(3‐36) to the Y_2_ receptor was ~15 times higher than to the Y_1_ receptor (Gehlert et al., 1996); therefore, it remains possible that in our RMA, NPY‐(3‐36) activated the Y_1_ receptor at higher concentrations.

All known subtypes of NPY receptors are coupled to the heterotrimeric G_i/o_ protein (Alexander, Christopoulos et al., 2021), and NPY treatment has been shown to oppose G_s_‐mediated vasodilation (Abel & Han, 1989; Gulbenkian et al., 1992), consistent with the classical model of Gα_i/o_ signalling. Gα_i/o_ is not directly linked to elevation of [Ca^2+^]_*i*_ and VSM contraction, but the Gβγ subunit can dissociate upon receptor activation and directly couple to PLCβ2 to mobilise intracellular Ca^2+^ (Camps et al., 1992; Smrcka & Sternweis, 1993), leading to vasoconstriction akin to U‐73122‐ and PTX‐sensitive constriction by activation of the G_i/o_‐coupled A_1_ adenosine receptor (Hansen et al., 2003). To investigate the involvement of the Gβγ subunit, we utilised gallein, a small‐molecule inhibitor that binds to Gβγ's interaction hotspot with downstream protein targets such as PLC (Lehmann et al., 2008). From the abolition of vasoconstriction, intracellular Ca^2+^ events and depolarization by both gallein and U‐73122, we concluded that gallein disrupted the Y_1_ receptor–Gβγ–PLCβ2 coupling. The inhibition of NPY‐induced vasoconstriction by gallein was concentration dependent, with maximal block achieved at 100 μM (Figure S5A), consistent with findings from Meens et al. (2012) showing that concentration‐dependent inhibition of CGRP‐mediated relaxation by gallein was maximal at 100 μM. In our hands, 100 μM gallein did not significantly block major signalling pathways linked to vasoconstriction (Figure S5), supporting its specificity in inhibiting NPY‐activated vasoconstriction and Ca^2+^ events. Previous studies have shown that Y_1_ receptor activation stimulates PLC activity (Robidoux et al., 1998) and that binding of NPY to the Y_1_ receptor was inhibited by U‐73122 (S. L. Parker et al., 1998), implying functional and physical coupling between the Y_1_ receptor and PLC; our findings demonstrate Gβγ as the missing link in this signalling pathway. However, one caveat in utilising U‐73122 to block PLC is the potential for off‐target effects, namely, in inhibiting the sarcoplasmic/endoplasmic reticulum Ca^2+^‐ATPase (SERCA) (Hollywood et al., 2010) and depolarization‐induced constriction (Garland et al., 2017). To minimise any potential for off‐target effects, we optimised incubation so U‐73122 inhibited PE‐ but not depolarization‐induced vasoconstriction (Figure S6).

PTX has traditionally been used specifically to inhibit the G_i/o_‐coupled receptors, by ADP‐ribosylating the Gα_i/o_ subunit and thus preventing nucleotide exchange, keeping Gβγ sequestered in an inactive state (Camps et al., 1992; Katada & Ui, 1982). There are precedents suggesting NPY vasoconstriction activated by NPY (Andriantsitohaina et al., 1990) or other G_i/o_‐coupled receptors (Hansen et al., 2003) is sensitive to PTX. However, in our hands, PTX did not significantly inhibit NPY‐induced vasoconstriction. Our control data suggest that PTX did not sufficiently permeate the smooth muscle layers to access SMC G_i/o_ proteins, based on immunohistochemical staining with PTX which was restricted to the adventitia, but not evident in SMC layers (Figure S3C,D).

NPY has previously been reported to depolarize rabbit cerebral (Abel & Han, 1989), rat cerebral (Xia et al., 1992) and rat mesenteric (Prieto et al., 1997) arterial VSM. There is also evidence suggesting that NPY directly inhibits K_Ca_ channels in isolated VSM cells (Xiong & Cheung, 1995), further depolarizing the membrane. In addition to slow, graded depolarization of RMA VSM by NPY, we also report the development of transient, AP‐like spikes at high NPY concentrations (10–100 nM). Arterial VSM cells are usually electrically quiescent under basal conditions, but we previously showed that transient spikes can be induced by vasoconstrictor agents upon loss of NO synthesis, which is linked to development of vasospasm (Smith et al., 2020). Therefore, elevated levels of NPY, especially in patients with endothelial dysfunction, could pre‐dispose the patients to develop microvascular vasospasm. Our previous work using RMA indicated that following inhibition of NOS, PE‐induced VSM depolarization beyond approximately −40 mV triggered AP‐like spikes, which was attributed to Ca^2+^ influx through both T‐type and L‐type VGCCs (Smith et al., 2020). Thus, the transient AP spikes we observed in the current study reflect changes in intracellular Ca^2+^ levels through VGCC‐mediated Ca^2+^ influx, as NPY also activated synchronised Ca^2+^ flashes in non‐myogenic RMA at a similar frequency (1.1 ± 0.2 Hz AP vs. 0.98 ± 0.15 Hz Ca^2+^ flashes) and increased the frequency of flashes in myogenic RCA.

A number of groups have previously measured changes in global [Ca^2+^]_*i*_ in cultured VSM cells (Jacques et al., 2000; Mihara et al., 1989) or intact RMA (Prieto et al., 2000) in response to NPY challenge, but to our knowledge, we are the first to quantify NPY‐activated Ca^2+^ waves (Herring et al., 2019) and synchronised Ca^2+^ flashes in intact arteries. Upon NPY stimulation, the intracellular Ca^2+^ release manifested as Ca^2+^ waves that propagate throughout individual VSM cells. After 87 ± 14 s, the waves subsided or were masked and evolved into synchronised Ca^2+^ flashes, likely representing Ca^2+^ propagating and signalling through gap junctions that physically and electrically couple neighbouring VSM cells (Borysova et al., 2018; Dora et al., 2022). The capability of NPY to initiate such electrical coupling clearly demonstrates the potential of local NPY release to evoke and spread vasospasm throughout an artery.

As only male rats were used in the present study, to limit inter‐sex variability, the conclusions generated from our study may not apply to females, especially as NPY expression in rats is sexually dimorphic (Urban et al., 1993).

A myriad of evidence in the literature indicates that high NPY levels and receptor activation are detrimental to the cardiovascular system and that inhibitory modulation of NPY activity may be beneficial. For example, a rat myocardial infarction (MI) model with global NPY knockout exhibited smaller infarct size and lower cardiomyocyte apoptosis compared with WT (Huang et al., 2019). However, a recent knockout study demonstrated that NPY^−/−^ mice had more severe MI, fibrosis, inflammation and cardiac dysfunction, which were reversed with exogenous NPY, revealing potential cardioprotective effects of certain NPY pathways (Qin et al., 2022). Indeed, NPY activity, primarily through Y_2_ receptor activation, is linked to angiogenesis, cardiomyocyte differentiation, EC proliferation and macrophage polarisation into the reparative M2 phenotype (Lee et al., 2003; Qin et al., 2022; Saraf et al., 2016).

Given the complex nature of NPY signalling in various cell systems, development of therapeutic options requires precise targeting and specificity to cell types and receptor–effector coupling, rather than global ablation of NPY activity. Our results indicate the potential of targeting Y_1_ receptor to limit sympathetic‐driven, NPY‐induced microvascular dysfunction, but side effects are possible given that Y_1_ receptor also regulates cardiomyocyte contractility (Heredia et al., 2005) and hypertrophy (Nicholl et al., 2002). Downstream of Y_1_ receptor activation, the Gβγ subunit may be a potentially attractive target, but global targeting of the ubiquitously expressed Gβγ might give rise to undesired side effects, as Gβγ also regulates adenylyl cyclases, PI_3_K and ion channels (Smrcka, 2008). Furthermore, inhibiting the reassociation of Gβγ to Gα could disrupt G protein cycling, thus impacting a great range of GPCR function. Genetic down‐regulation of individual β and γ isoforms is associated with significant complications, such as reducing the expression of other G protein subunits or affecting the stability, membrane localization and function of various receptor signalling complexes (reviewed in Dupre et al. (2009); Khan et al. (2013)). To this end, small‐molecule Gβγ inhibitors gallein and M119 were developed, circumventing the need to ablate Gβγ expression; the authors have shown that they blocked Gβγ interactions with PLCβ2–3 and PI_3_K, but Gα‐mediated signalling was intact, thus preserving G protein cycling and stability (Bonacci et al., 2006; Lehmann et al., 2008). In the past decade, a few preclinical models of Gβγ targeting using gallein or M119 have been developed, including of chronic inflammation diseases, pain and heart failure (reviewed in Campbell & Smrcka, 2018). Uncoupling of VSM Gβγ–PLCβ2 interaction with these small‐molecule inhibitors may, therefore, represent a potential therapeutic avenue in ameliorating NPY‐related microvascular dysfunction.

In this study, we characterised the mechanism of NPY‐induced vasoconstriction, presenting the Gβγ subunit as the missing link between VSM Y_1_ receptor activation and Ca^2+^ mobilisation and membrane depolarization. Importantly, we show that NPY is able to elicit synchronised VSM Ca^2+^ flashes and AP spikes, which would be predicted to contribute to coronary microvascular dysfunction during periods of stress‐induced sympathetic activity, especially in patients with poor endothelial function.

## AUTHOR CONTRIBUTIONS

JinHeng Lin and Kim Dora designed the study. JinHeng Lin, Lauren Scullion and Christopher Garland performed the experiments. JinHeng Lin, Lauren Scullion, Christopher Garland and Kim Dora analysed the data. JinHeng Lin, Christopher Garland and Kim Dora wrote the manuscript. All authors have read the manuscript and given written consent for publication.

## CONFLICT OF INTEREST STATEMENT

The authors declare no conflict of interest.

## DECLARATION OF TRANSPARENCY AND SCIENTIFIC RIGOUR

This Declaration acknowledges that this paper adheres to the principles for transparent reporting and scientific rigour of preclinical research as stated in the BJP guidelines for Natural Products Research, Design and Analysis, Immunoblotting and Immunochemistry and Animal Experimentation and as recommended by funding agencies, publishers and other organisations engaged with supporting research.

## Supporting information


**Figure S1:** Myogenic tone in rat coronary artery (RCA). Summary of myogenic tone above basal tension in coronary arteries without pharmacological intervention (control), or in the presence of L‐NAME; NS 6180 + apamin; or the combination of all three drugs. n is indicated in parentheses.
**Figure S2:** Negative control for immunohistochemical staining of Y_1_ receptor, NPY and tyrosine hydroxylase. Representative (n = 4 for rat mesenteric artery (RMA) (A) and n = 3 for rat coronary artery (RCA) (B)) immunohistochemistry labelling for Alexa Fluor 488, 546, and 647 with the same laser settings as images acquired for Figure 2c and f, with the primary antibodies omitted. Nuclei were labelled in blue.
**Figure S3:** The effects of pertussis toxin on NPY‐induced vasoconstriction in rat mesenteric artery (RMA). (A) Concentration‐response curves of NPY‐induced constriction in RMA pre‐treated with L‐NAME (L‐N), with or without PTX (100 ng·ml^−1^ for 1 h or 300 ng·ml^−1^ for 3 h). Vasoconstriction was normalised to the response elicited by 45 mM K^+^ Krebs solution. n is indicated in parentheses. (B) Summary of the E_max_ elicited by NPY from experiments in (A). Kruskal‐Wallis test, with Dunn's multiple comparisons test against the L‐N group was performed. (C, D) Representative (n = 5 for each group) immunohistochemistry labelling for PTX throughout different layers of arteries pre‐treated with 300 ng·ml^−1^ PTX (C) or arteries without any PTX exposure (D). Nuclei were labelled in blue.
**Figure S4:** Gallein inhibits NPY‐induced vasoconstriction independently of L‐NAME in rat mesenteric artery (RMA) (A, B) and rat coronary artery (RCA) (C, D). (A, C) Concentration‐response curves of NPY induced constriction in arteries with or without pre‐treatment with gallein (100 μM, 30 min). Vasoconstriction was normalised to the response elicited by 45 mM K^+^ Krebs solution. n is indicated in parentheses. (B, D) Summary of the E_max_ elicited by NPY from experiments in (A,C). Mann–Whitney test was performed for (B), and unpaired t‐test was performed for (D).
**Figure S5:** Gallein does not inhibit depolarization‐induced and Gq‐coupled vasoconstriction, Ca^2+^ flashes, and myogenic tone. (A) Effect of varying concentrations of gallein (10–100 μM) on NPY‐induced vasoconstriction in rat mesenteric artery (RMA). n is indicated in parentheses. (B) Summary of vasoconstriction activated by 45 mM K^+^ Krebs solution before and after incubation with 100 μM gallein. n = 10 for RMA, n = 6 for rat coronary artery (RCA). (C) Summary of RMA vasoconstriction activated by 1–3 μM phenylephrine (PE) before and after incubation with 100 μM gallein. n = 5. (D) Simultaneous measurements of isometric tension and Ca^2+^ flashes in response to PE in RMA pre‐treated with 100 μM gallein. The traces for whole field Ca^2+^ flashes are magnified, corresponding to sections indicated by red boxes on the tension trace. n = 3. (E) Summary of myogenic tone above basal tension in RCA without pharmacological intervention (control), or in the presence of L‐NAME, with or without the presence of 100 μM gallein. n is indicated in parentheses.
**Figure S6:** The effects of U‐73122 on PLC‐dependent and depolarization induced vasoconstriction. Summary of vasoconstriction activated by 1–3 μM PE (A) or 45 mM K^+^ Krebs solution (B), before and after incubation with 3 μM U‐73122. Vasoconstriction was normalised to the first challenge with PE/45 mM K^+^, before exposure to U‐73122. n = 8 for RMA, n = 7 for RCA.
**Figure S7:** Representative images of Calbryte 520 AM‐loaded SMC in RMA and RCA mounted on wire myograph. The field of view and regions of interest of each panel correspond to representative Ca^2+^ imaging experiments (a‐f) in Figure 4.

## Data Availability

The data that support the findings of this study are available from the corresponding author upon reasonable request. Some data may not be made available because of privacy or ethical restrictions.
