# Supplementary material for: Gβγ subunit signalling underlies neuropeptide Y‐stimulated vasoconstriction in rat mesenteric and coronary arteries
Source: Br J Pharmacol. 2023 Aug 8;180(23):3045–58. doi: 10.1111/bph.16192 (PMC10953346; doi:10.1111/bph.16192)
Supplement: Supplementary file 1 — Figure S1: Myogenic tone in rat coronary artery (RCA). Summary of myogenic tone above basal tension in coronary arteries without pharmacological intervention (control), or in the presence of L‐NAME; NS 6180 + apamin; or the combination of all three drugs. n is indicated in parentheses. Figure S2: Negative control for immunohistochemical staining of Y1 receptor, NPY and tyrosine hydroxylase. Representative (n = 4 for rat mesenteric artery (RMA) (A) and n = 3 for rat coronary artery (RCA) (B)) immunohistochemistry labelling for Alexa Fluor 488, 546, and 647 with the same laser settings as images acquired for Figure 2c and f, with the primary antibodies omitted. Nuclei were labelled in blue. Figure S3: The effects of pertussis toxin on NPY‐induced vasoconstriction in rat mesenteric artery (RMA). (A) Concentration‐response curves of NPY‐induced constriction in RMA pre‐treated with L‐NAME (L‐N), with or without PTX (100 ng·ml−1 for 1 h or 300 ng·ml−1 for 3 h). Vasoconstriction was normalised to the response elicited by 45 mM K+ Krebs solution. n is indicated in parentheses. (B) Summary of the Emax elicited by NPY from experiments in (A). Kruskal‐Wallis test, with Dunn's multiple comparisons test against the L‐N group was performed. (C, D) Representative (n = 5 for each group) immunohistochemistry labelling for PTX throughout different layers of arteries pre‐treated with 300 ng·ml−1 PTX (C) or arteries without any PTX exposure (D). Nuclei were labelled in blue. Figure S4: Gallein inhibits NPY‐induced vasoconstriction independently of L‐NAME in rat mesenteric artery (RMA) (A, B) and rat coronary artery (RCA) (C, D). (A, C) Concentration‐response curves of NPY induced constriction in arteries with or without pre‐treatment with gallein (100 μM, 30 min). Vasoconstriction was normalised to the response elicited by 45 mM K+ Krebs solution. n is indicated in parentheses. (B, D) Summary of the Emax elicited by NPY from experiments in (A,C). Mann–Whitney test was performed for (B) [file BPH-180-3045-s001.pdf]

Supporting Information

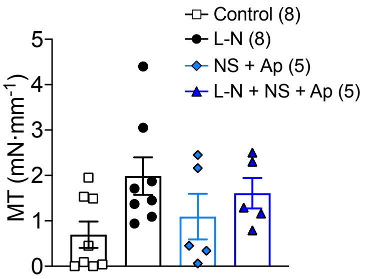

Figure S1: Myogenic tone in rat coronary artery (RCA). Summary of myogenic tone above basal tension in coronary arteries without pharmacological intervention (control), or in the presence of L-NAME; NS 6180 + apamin; or the combination of all three drugs. n is indicated in parentheses.

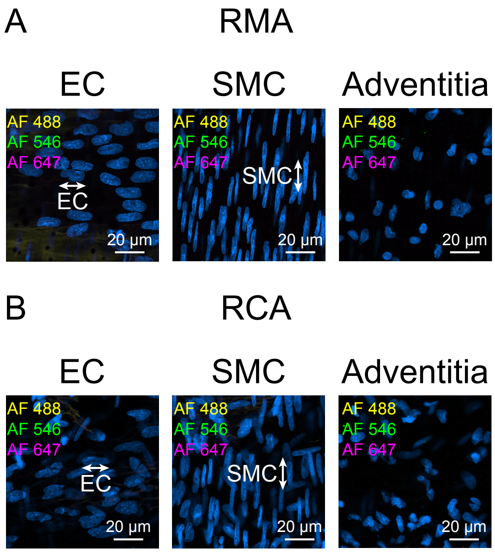

Figure S2: Negative control for immunohistochemical staining of Y_1 receptor, NPY and tyrosine hydroxylase. Representative (n = 4 for rat mesenteric artery (RMA) (A) and n = 3 for rat coronary artery (RCA) (B)) immunohistochemistry labelling for Alexa Fluor 488, 546, and 647 with the same laser settings as images acquired for Figure 2c and f, with the primary antibodies omitted. Nuclei were labelled in blue.

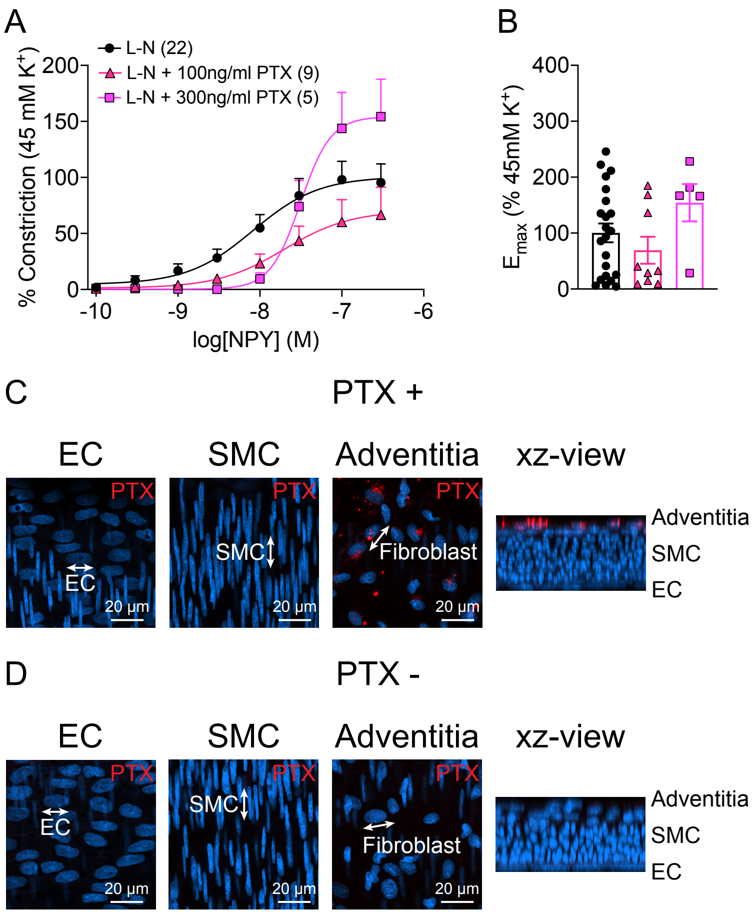

Figure S3: The effects of pertussis toxin on NPY-induced vasoconstriction in RMA. (A) Concentration-response curves of NPY-induced constriction in RMA pre-treated with L-NAME (L-N), with or without PTX (100 $ng{\cdot}ml^{-1}$ for 1 h or 300 $ng{\cdot}ml^{-1}$ for 3 h). Vasoconstriction was normalised to the response elicited by 45 mM K^+ Krebs solution. n is indicated in parentheses. (B) Summary of the E_{max} elicited by NPY from experiments in (A). Kruskal-Wallis test, with Dunn's multiple comparisons test against the L-N group was performed. (C, D) Representative (n = 5 for each group) immunohistochemistry labelling for PTX throughout different layers of arteries pre-treated with 300 $ng{\cdot}ml^{-1}$ PTX (C) or arteries without any PTX exposure (D). Nuclei were labelled in blue.

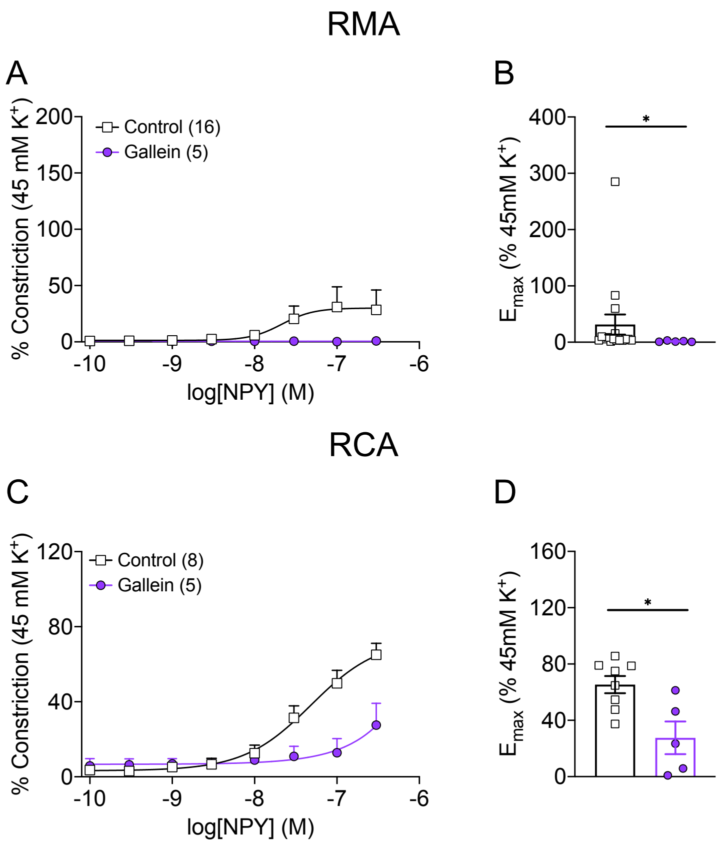

Figure S4: Gallein inhibits NPY-induced vasoconstriction independently of L-NAME in rat mesenteric artery (RMA) (A, B) and rat coronary artery (RCA) (C, D). (A, C) Concentration-response curves of NPY-induced constriction in arteries with or without pre-treatment with gallein (100 μM, 30 min). Vasoconstriction was normalised to the response elicited by 45 mM K^+ Krebs solution. n is indicated in parentheses. (B, D) Summary of the E_{max} elicited by NPY from experiments in (A, C). Mann-Whitney test was performed for (B), and unpaired t-test was performed for (D).

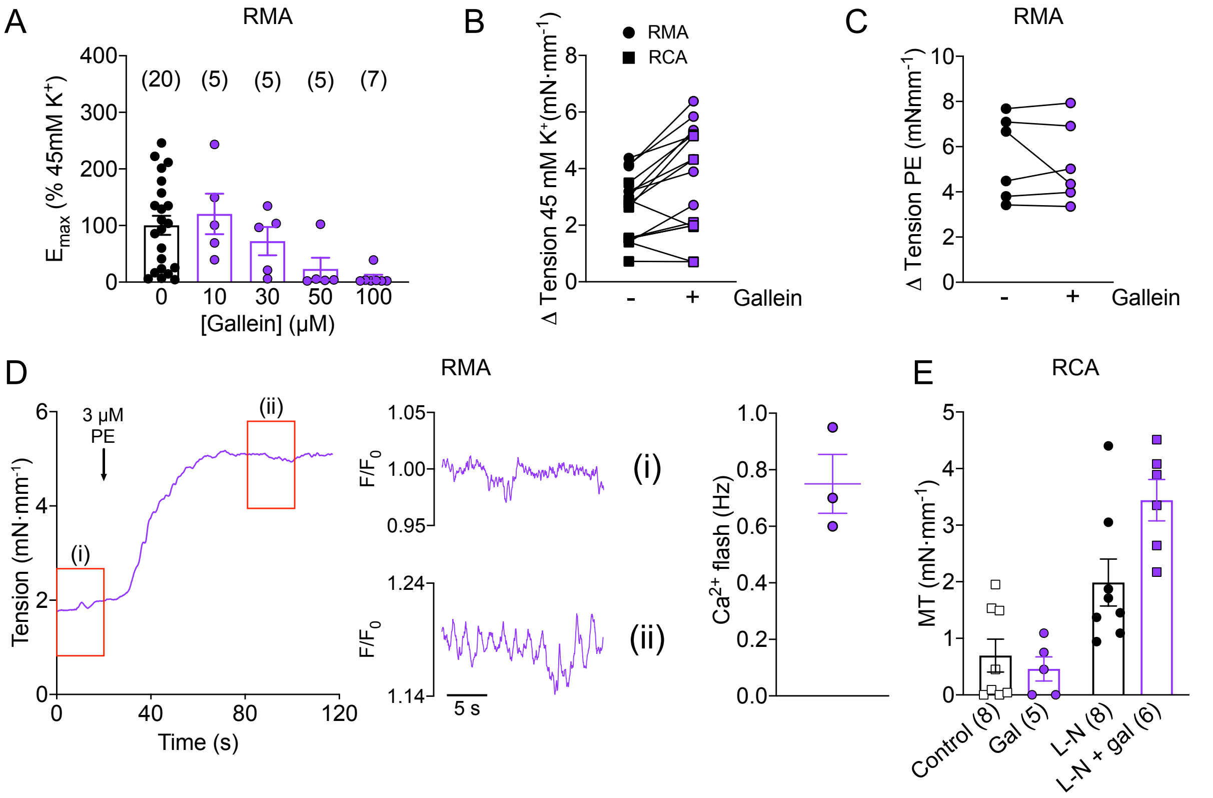

Figure S5: Gallein does not inhibit depolarization-induced and G_q-coupled vasoconstriction, Ca^{2+} flashes, and myogenic tone. (A) Effect of varying concentrations of gallein (10-100 μM) on NPY-induced vasoconstriction in rat mesenteric artery (RMA). n is indicated in parentheses. (B) Summary of vasoconstriction activated by 45 mM K^+ Krebs solution before and after incubation with 100 μM gallein. n = 10 for RMA, n = 6 for rat coronary artery (RCA). (C) Summary of RMA vasoconstriction activated by 1-3 μM phenylephrine (PE) before and after incubation with 100 μM gallein. n = 5. (D) Simultaneous measurements of isometric tension and Ca^{2+} flashes in response to PE in RMA pre-treated with 100 μM gallein. The traces for whole field Ca^{2+} flashes are magnified, corresponding to sections indicated by red boxes on the tension trace. n = 3. (E) Summary of myogenic tone above basal tension in RCA without pharmacological intervention (control), or in the presence of L-NAME, with or without the presence of 100 μM gallein. n is indicated in parentheses.

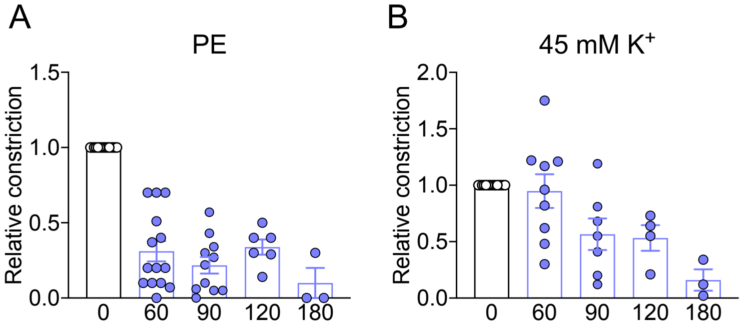

Figure S6: The effects of U-73122 on PLC-dependent and depolarization-induced vasoconstriction. Summary of vasoconstriction activated by 1-3 μM PE (A) or 45 mM K^+ Krebs solution (B), before and after incubation with 3 μM U-73122. Vasoconstriction was normalised to the first challenge with PE/45 mM K^+, before exposure to U-73122. n = 8 for RMA, n = 7 for RCA.

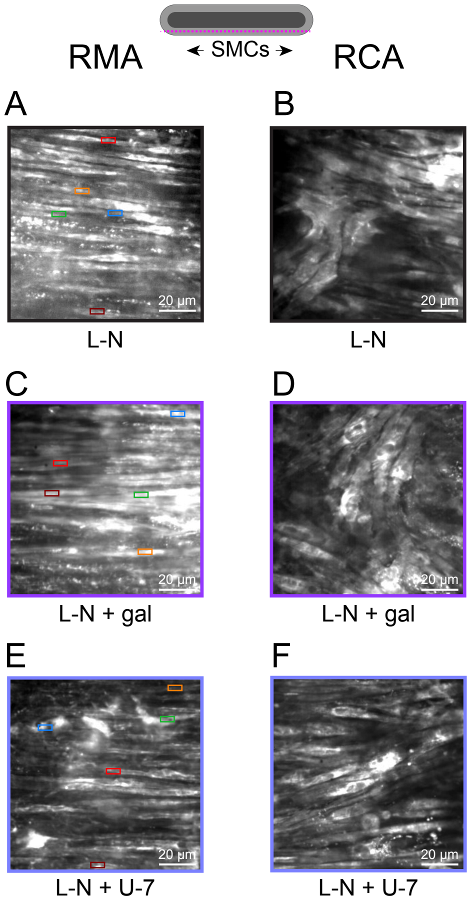

Figure S7: Representative images of Calbryte 520 AM-loaded SMC in RMA and RCA mounted on wire myograph. The field of view and regions of interest of each panel correspond to representative Ca^{2+} imaging experiments (a-f) in Figure 4.
